# An assessment of subject-comprehension and subject-command attained through digital learning platforms

**DOI:** 10.1007/s44217-022-00020-z

**Published:** 2022-12-12

**Authors:** Nazish Shahid

**Affiliations:** Department of Mathematics, Forman Christian College (A Chartered University), Lahore, Pakistan

**Keywords:** Digital mode of education, Insufficient subject understanding, Machine learning, Uniform access to digital coaching platforms, Data visualization, Interaction-Regression

## Abstract

A synthesized investigation, employing graphical and analytical approach, has been conducted to examine inadequacy of electronic education and limitations posed by transformative mode of learning from students’ perspective. Moreover, the breadth of subject understanding through digital mode and students’ preference for physical or electronic mode of learning in the future were examined. A descriptive analysis was executed through R programming for the obtained numeric-characteristic statistics. For computational analysis of the data to determine proportion of deteriorating virtual-assessment performance attributed to conditioned subject-command, a machine learning approach of interaction-regression is adopted. It is implied through the obtained results that a majority of students felt discontented at not being able to achieve optimized learning outcomes post-virtual-attendance of study programs. It is also concluded that blended influence of online learning and partial subject-command resulted in insufficient assessment performance. Additionally, the current study highlights the importance of need-based adaptations to facilitate automated mode of learning and virtual platforms’ uniform access to students.

## Introduction

### Context of the study

Among scores of changes brought on by Covid-19 pandemic in about every field of life, deliverance of education hinged on an abrupt adaptation to an electronic mode of education. Undoubtedly, an infinite breadth of man’s intellectual capacity and his inherent continuance-predisposition allowed the world to see phenomenal practices put together to ensure uninterrupted delivery of education. Despite the innovations of academic world to continue schooling in these challenging times, numerous aspects of education seemed to be compromised and called for efforts to address the related concerns.

A transition in the mode of instruction from a classroom to a digital platform necessitates an act of perceptive remodeling of a system structure. An uninformed adjustment of central blocks of this structure will cause restraining the scope of comprehensive scholastic goals. It is essential to pre-determine the impact of remote education on all components of a learning system. Moreover, in order to allow coordinated evolvement of all sections of a virtual educational construct, it is crucial to encompass the spectrum of associated problems and their role in manipulating the educational dynamics. An improved framework of virtual or hybrid learning system is intended to aid the purpose of uninterrupted education provision in the light of these considerations. A virtual educational framework mainly comprises of digital infrastructure, operative mechanism, regulating policies, subject content and presence of physical characters making use of this system. Lack in output of even one feature of the framework can impede congenial working of all the factors contributing to steady functioning of the system. In view of this conception, both learners and instructors’ satisfaction with digital learning and transmitting episodes, respectively, will assist in the improvement of standard hybrid/distance educational construct. Moreover, uniform access to digital educational infrastructure, inclusive virtual sessions, trainings to operate in virtual environment, exchange of technology types in conjunction with subject content and incorporation of receptive digital outlook can further contribute to improving the digital scholarly framework.

### Literature review

A multitude of scholarly efforts have been conducted to highlight and address the challenges of digital or remote education [1–4, 7, 9, 13, 16, 18, 19, 21, 23, 24, 29, 34, 37]. One significant problem related to implementation of digital educational system is the use of one-size fits all approach in changing between and across modes of delivery. This approach is contingent upon the idea of internal content being converted into a form deemed suitable for external delivery [12]. It is further reported that this method heightens the barriers for students feeling or experiencing isolation. This insight therefore presents another type of learner to consider in the planning and implementation of learning activities online. Another challenge in relation to transformation of mode of education is technology incorporation in teaching and learning practices. Orlando and Attard [22] observed that the use of technology in teaching varies, depending upon its type to be implemented and on the content being taught. The technology improvisation and adaptation in accordance with available resources and content bearings, therefore, present further review points for the improvement of virtual learning experience. Kirkwood and Price [14] emphasized on re-evaluating the assumption that technologies can always enhance the knowledge and student engagement. In view of an online framework development challenges, Graham and Misanchuk [8] pointed to an issue of slackening focus on content delivery in online study programs. The act of accomplishing online tasks and collaborative learning activities, that can cause individual differences to be highlighted, is rather paid more attention. Shahid [28] conducted a survey-based study to exhibit an inadequacy of students to perform to their ceiling potential in virtual-assessments due to slow adaptation to an online habitat. Davidson and Jaques and Salmon [6, 11] reported some problems experienced by students in an online learning system such as being out of sorts with technology handling, perception of inequity in collaborative tasks and conditioned peer interaction. Jaques and Salmon, Little-Wiles and Naimi, Rucker and Downey, Schmidt et al, Thorsteinsson [11, 17, 25, 26, 31] deliberated on the issues of teaching staff feeling apprehensive of the task of an online delivery due to not being suitably equipped with virtual platforms’ use.

In recent times, the use of machine learning techniques in an efficient decision process has demonstrated tremendous success in the field of research and education. Ciolacu et al [5] conducted an analysis based on neural networks and support vector machine to assess the learning quality. Tong and Srivastava [32] maintained that students’ learning ability and academic performance are observed to be significantly improved with the application of intelligent distance online education based on cloud computing. Wang [35] reported that combining a development trend, the Internet of things, with a mature business model will promote the industrialization of e-education, and improve the realization mechanism of Chinese distance education process. In consonance with these developments, we intend to apply machine-learning algorithm to examine students’ perceived command on subject-content through virtual coaching programs.

Sebastianelli et al [27] examined the significance of subject-comprehension and satisfaction in an online learning program. The research findings suggested that content-command was the only significant factor affecting the perceived learning. One such component related to digital educational impediments is loss of certainty on the part of a student about comprehensive command of subject-knowledge [28]. An absence of interactive-learning opportunities and motivating academic environment in addition to technological straits of e-platforms conditioned students’ radical understanding of a subject.

The above-mentioned research endeavors have pointed to an obligation to address the issues that virtual experience of students is causing them to be more isolated, and both students and teachers need to be tech-trained for optimized experience in a digital environment. It was demonstrated through an analysis of a huge data that technology incorporation in instruction methods did not necessarily enhance students’ understanding of a subject. Moreover, in a study of developing a requisite digital learning framework, the problem of virtual collaborative activities promoting individual differences among students was discussed. The obstacles faced by students during virtual assessments and inadequate tech-learning sessions were also mentioned. Additionally, the strong relevance of subject comprehension and perceived learning was communicated. However, the impact of online learning on students’ comprehensive material understanding and the subsequent virtual-testing performance was not assessed. In view of the proposition of recognized universities to establish virtual/distance learning as a prime medium of instruction to ease education access to remote areas, it is essential to weigh the significant connection of e-education and learning graph of students. It is equally important to fully encompass the reasons for hampered virtual assessment-performance of students [28] by analyzing the factors involved in the process of digital learning. The current study aims at highlighting the disposition of learners in regard to availing online educational programs, the extent to which they are sure of subject understanding, and their preference for physical or electronic mode of learning in the future. Moreover, a computational approach has been adopted to infer from the data that students’ command on an online-instructed subject seems to have slackened.

## Data formation

**Fig. 1:**
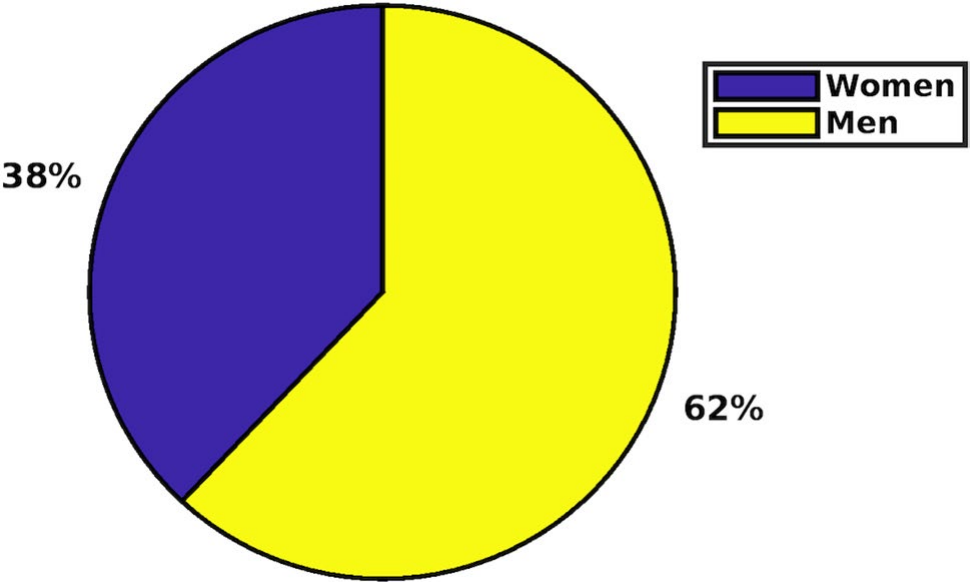
Gender Participation Classification

The current study has been prepared based on the data collected from 200 senior Bachelor of Science and Arts students of local universities during the month of November 2021. The participants were selected using a method of simple random sampling to reduce sampling bias and to have an accurate estimation of data disposition. The survey questions’ link was shared with elected participants on an online learning platform Moodle with a clear set of instructions about response formation and time frame. Moreover, the Google survey form integrated with Google spreadsheet was transformed in an excel spreadsheet for an organized analysis of the obtained results. The percentage for participation of men and women respondents of age bracket 20–27 is displayed in Fig. 1.

**Fig. 2:**
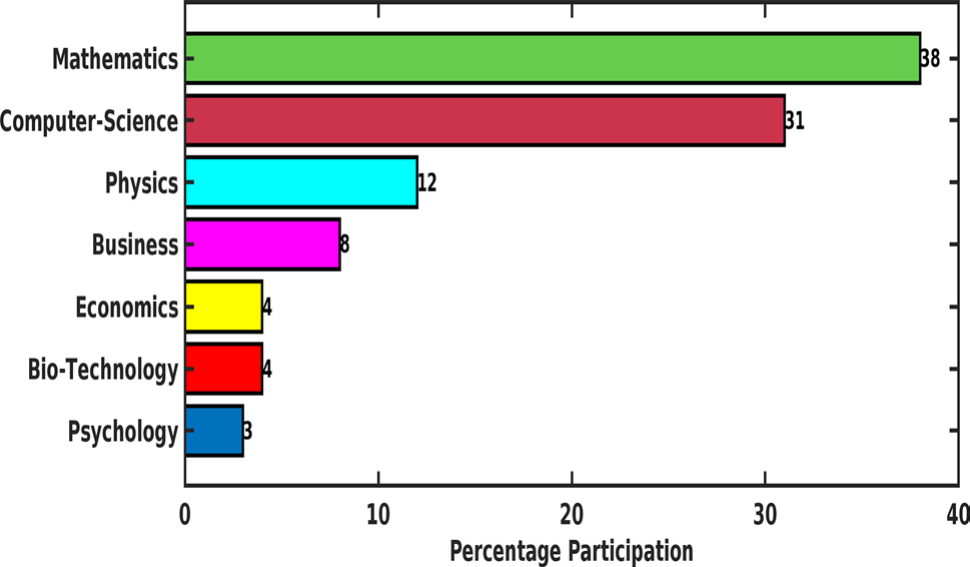
Percentage Participation of Programs in the Survey

Also, students’ representation from different programs is reported in Fig. 2.

## Research methodology

A consolidation of exploratory and descriptive research designs [30] has been employed to assess students’ grasp of subject-content through an online medium. To develop an exploratory part of a conceptual framework, a set of questions inquiring selected students’ response to availing virtual training programs was prepared. Moreover, the association of variables influencing the comprehension of subject matter through digital platforms was examined. Furthermore, in order to build up a descriptive narrative of the problem, a hypothesis of virtual-exam performance being influenced by conditioned subject-comprehension and subject-command was tested on the survey data.

To conduct an analysis on the data, a set of definite questions was designed to collect information about students’ comprehension and command for the courses taken online. In addition to subjective feedback of participants’ perceived understanding of subject-comprehension and subject-command, assessment scores of related subjects and their pre-requisites were collected to evaluate the phenomenon of content grasp. Furthermore, a quantitative association was developed between descriptive and statistical responses using a technique of cross validation [33].

The survey questions posed to the participants examined the ease with which they were able to access on-line classes & materials. The participants’ level of understanding the sciences and humanities subjects through virtual medium was appraised through numeric-characteristic count. Moreover, the candidates’ confidence of grasping a subject through screen-mechanism and their command on both the subject and assisting technology post-digital-participation [15] were assessed. Some data has also been generated to represent majority of participants’ inclination towards physical mode of attendance for study programs in the future. Primarily, the data was generated for the following questions:

Q1. Was it easier for you to access the virtual classes/materials?

Q2. Was it easier for you to understand the contents of a subject through online classes/virtual mediums?

Q3. Do you feel confident in having a good command on a subject after an online instruction program?

Q4. Did you get an equal command on both the subject and assisting technology through virtual participation?

Q5. Will you prefer/suggest an online mode of education to regular physical mode in the future?

In order to assess the compounded influence of subject-comprehension and subject-command attained through digital means on virtual-exam output, a computing analysis was administered. A blended regression approach was adopted to interpret the response of the following question:

Q6. Did subject-comprehension and subject-command, attained through digital platforms, affect the virtual-exam output?

R programming tools [10] have been used to visualize the captured data in the form of pie diagram and bar charts. An interaction-regression algorithm has been employed to probe the correlation between subject comprehension-command and improved virtual-exam performance.

## Results and discussion

The intrinsic purpose of the current analysis is to communicate scores of impediments that restricted students from availing optimized learning benefits of virtual learning platforms. In contrast to merely registering the general construct problems experienced by students in a transformed mode of learning, an effort is made to include the perspective of students to address and improve the general framework of a virtual system. In view of easing the elementary access to education by digitized means in the future, it is imperative to investigate the source of reservation of students towards virtual or hybrid learning. A comprehensive study of inclusive outlook reflecting on obstacles, interfering with maximized learning objective, is developed to bring changes in virtual learning design in accordance with these issues.

**Fig. 3:**
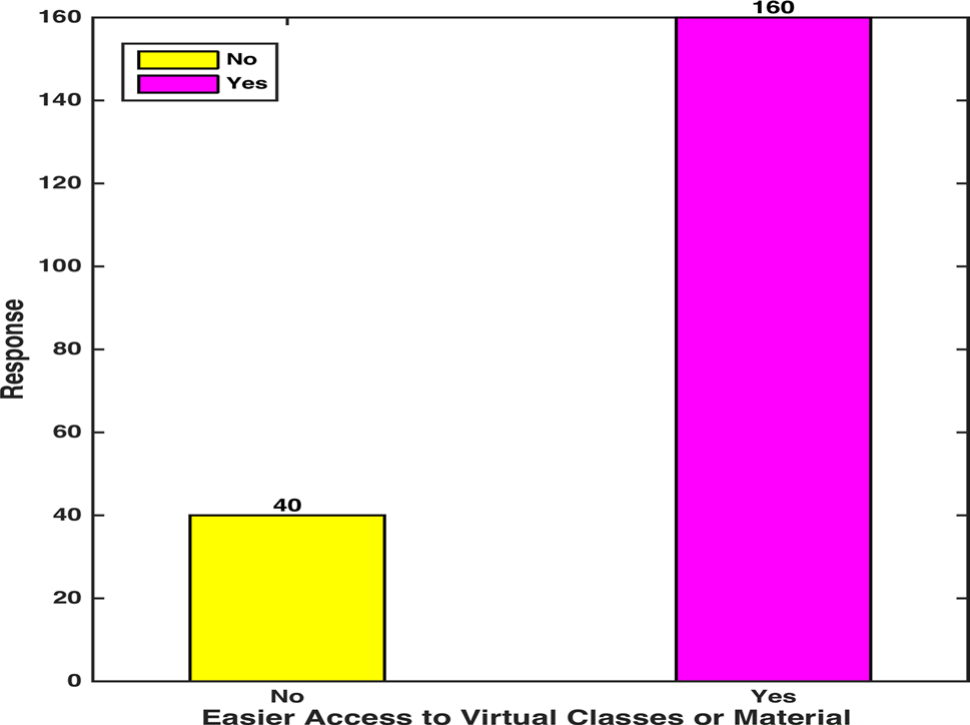
Virtual Class/Medium Access

The observations collected through visuals and computations have been discussed here. Figure 3 shows that 20 percent of the participants expressed their concerns about not accessing either the virtual classes or online subject-material. In this regard, multiple reasons for their foiled experiences such as Internet disconnectivity, power breakdown, bandwidth limitation and unavailability of certain foundational books in digital mode were reported [36]. Moreover, the time limit on free virtual platforms and some financial constraints prevented the students from fully benefitting the online practice. It can be observed that a fair percentage of candidates expressed their ease in accessing the online platforms and paraphernalia. However, the lack of uniform access points to an alarming widening gap between two classes of students from privileged and disadvantaged background.

**Fig. 4:**
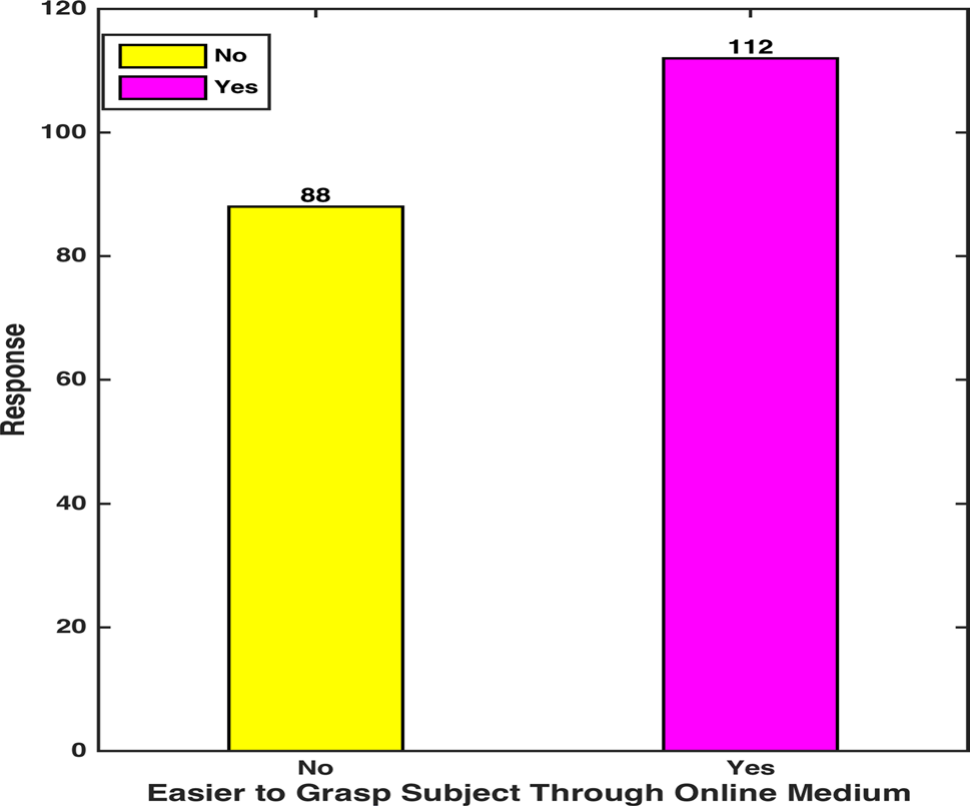
Subject-Comprehension through Digital Medium

Figure 4 points to a case where a sound proportion of the participants proclaimed their reluctance about not being able to fully perceive the contents of a subject through online instruction. Among 44 percent of the candidates doubting their subject command, the majority included online physical sciences programs’ attendants. One of the reported reasons was insufficient understanding of a topic or a method due to the absence of class-like interaction with an instructor [20]. A small percentage (0.2%) of the candidates attributed their ineffectual online experience to an inadequate knowledge of virtual class etiquettes.

**Fig. 5:**
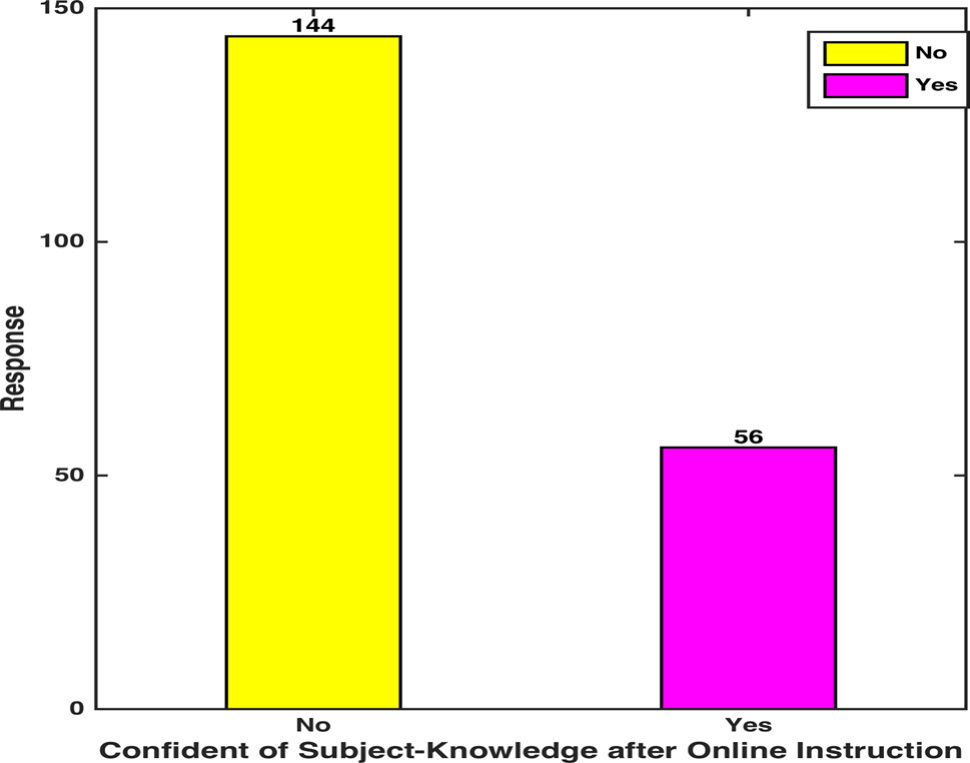
Subject-Knowledge Confidence

Figure 5 showcases an alarming majority of the participants asserting their lack of confidence in subject-knowledge in spite of achieving good grades. 72 percent of the total participants voiced their uncertainty about substantial gain from online learning programs. They reported developing an approach of cursory perceptiveness to glide them through subject assessments. However, the circumscribed understanding of basic ideas in foundational courses presented a significant challenge in comprehending the subsequent advanced material.

**Fig. 6:**
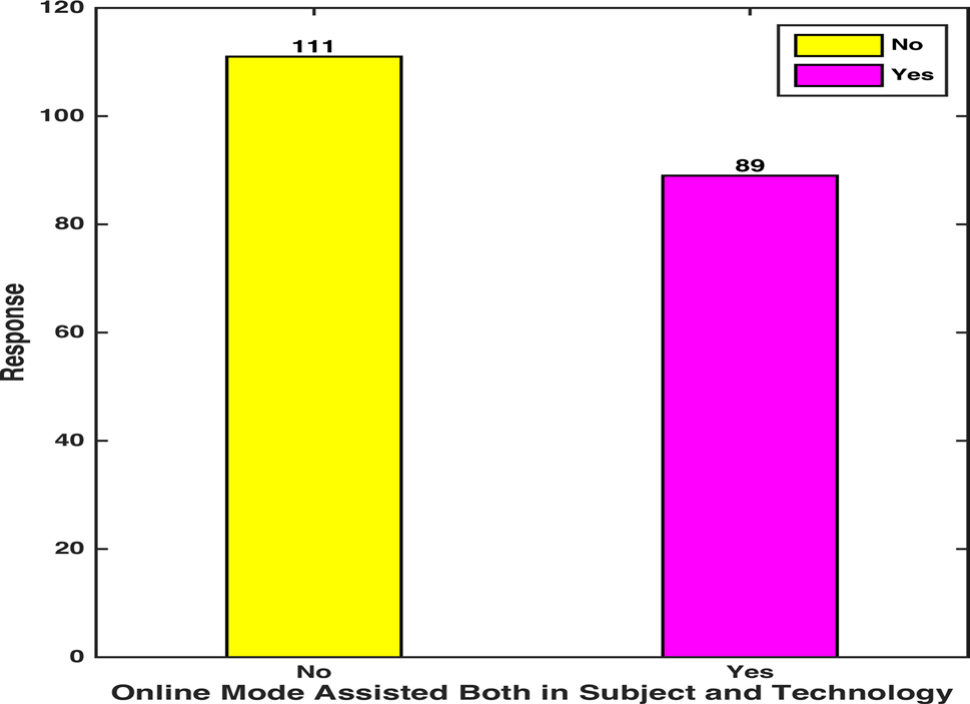
Subject and Technology Command

Figure 6 reflects on the dilemma of students of whether they were able to get simultaneous command on a subject and assisting technology, requisite to enter a symbolic/graphic response in a virtual assessment with visual clarity. A sound percentage (55.5%) of the participants manifested their ineffectiveness in gaining subject expertise along with the technological command, required to manage assessments and submissions. 12% of these participants conveyed that their focus of material comprehension deflected due to the time spent on understanding the technical details of e-platforms [12]. A considerable number of participants, particularly from mathematics and physics programs, expressed that their subject-comprehension was asymmetrically influenced by learning to demonstrate scientific symbols and graphic responses in different virtual platforms. Change of virtual mediums, compatible with different subjects, was also reported to interfere with an accurate solution presentation in electronic assessments, especially in physical sciences.

**Fig. 7:**
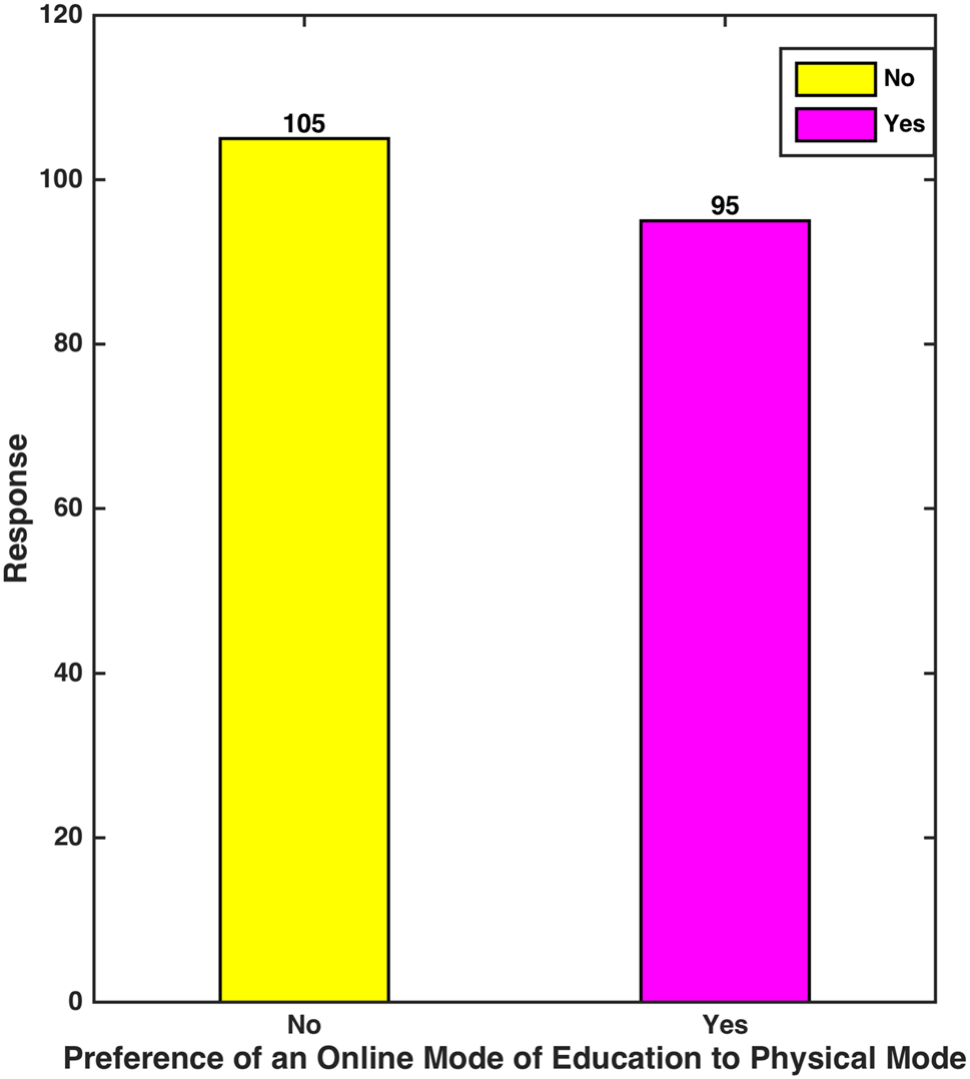
Mode of Education Preference

Figure 7 evidently speaks of 52.5% of the total participants’ preference of physical mode of education to virtual mode in the future. They expressed that physical class system offered them opportunities of an improved interaction with instructors and peers, a competitive ambience and a focused learning platform. Moreover, the problems of technological breakdown or an inability to access certain web-based programs were prevented.

### Regression analysis

A mixed-effects linear regression model has been adopted to determine the blended influence of digital subject-comprehension and subject-command on virtual-exam output. To conduct the impact simulation using statistical algorithm, virtual-exam output; subject-comprehension & subject-command have been chosen as response variable; predictor variables, respectively (Table 1).

**Table 1:** Classification of Variables

Type	Name	Notation
Response Variable	Virtual-Exam Output	R
Predictor Variables	Subject-Comprehension, Subject-Command	A, B

An interaction-model has been fit to analyze the data through machine learning mechanism, fitlm (–, ‘interactions’). A step-10 percolation was incorporated to improve the fit through addition or removal of terms. The structure and findings of this model have been displayed in Table 2.

**Table 2:** Linear Regression Measurements

Linear regression model: R = 1 + A + B + A.B				
Estimated coefficients:	Estimate	SE	tStat	p-Value
Intercept	0.13514	0.04204	3.2144	0.0015287
A	0.27915	0.060298	4.6296	6.6599e–06
B	0.86486	0.1054	8.2055	3.0015e–14
A.B	− 0.27915	0.12685	− 2.2006	0.028934

Number of observations: 200, Error degrees of freedom: 196

Root Mean Squared Error: 0.362

R-Squared: 0.486, Adjusted R-Squared 0.478

F-statistic vs. constant model: 61.8, p-value = 3.69e–28

It can be observed from p-values in Table 2 that the terms corresponding to intercept, subject-comprehension variable (A), subject-command variable (B) and their blended influence variable (A.B) are all significant at 5% significance level. An R-Squared estimation of 50% indicates that the virtual-exam output was substantially affected by digital subject-comprehension and conditioned subject-command. The potential fit of this model incorporating the simultaneous weight of cybernetic comprehension and command is represented through the significant p-value (= 0.028934 < 0.05). Moreover, a very small value of p for F-statistics indicates that the inclusion of predictors (A, B) and mixed-effects term (A.B) improves the model from an intercept-only model. The intercept-only model is attributed to no correlation between online exam output and online subject comprehension-command.

**Fig. 8:**
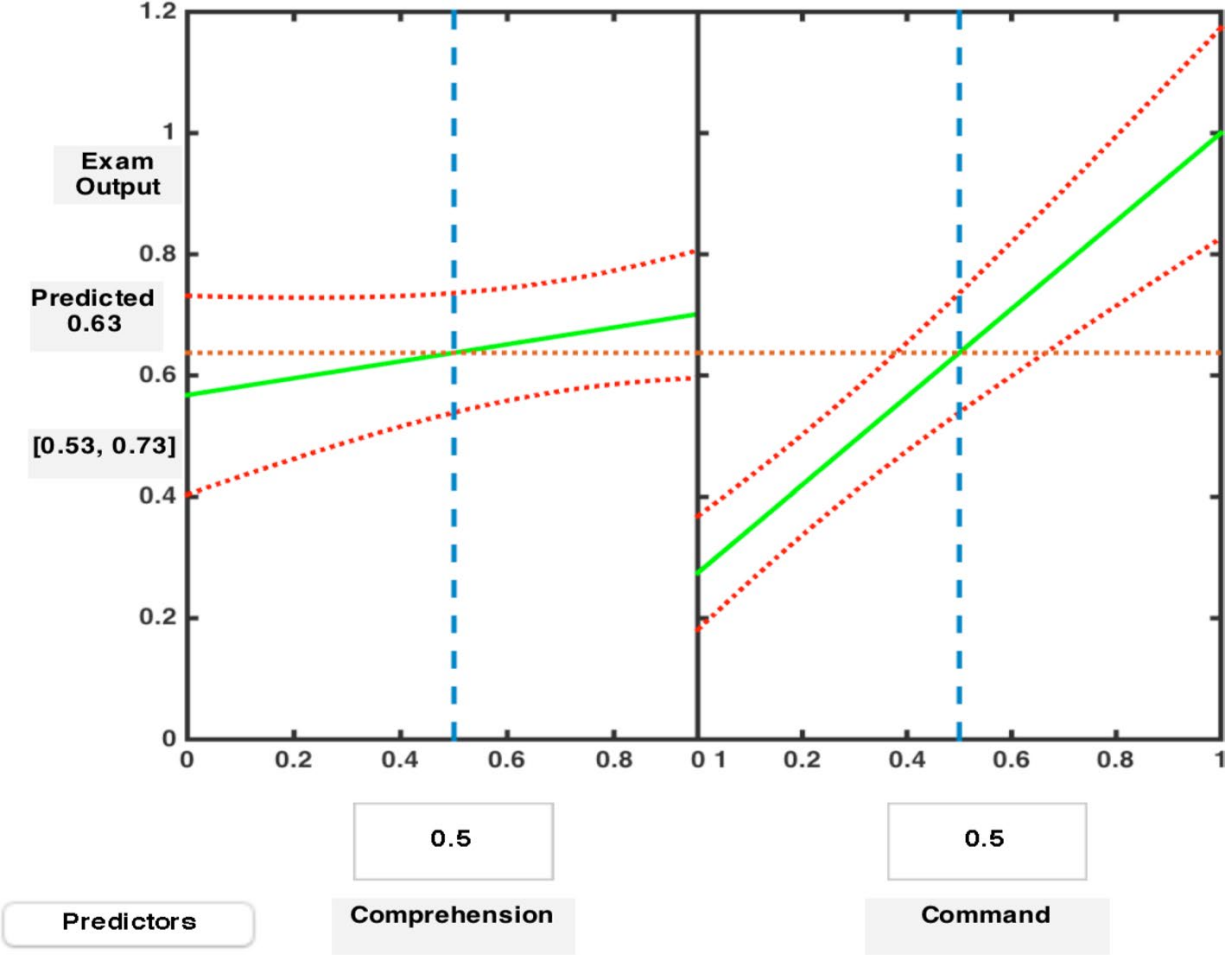
A Slice Plot for Comprehension-Command Variation

**Fig. 9:**
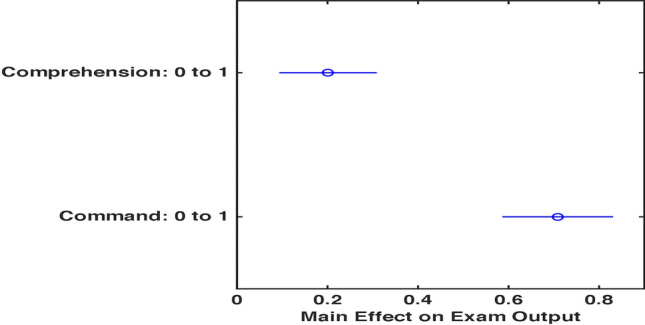
An Interaction Plot for Prediction

In order to understand the individual effects of online subject-comprehension and subject-command on virtual-exam output, we have constructed a slice plot (Fig. 8) representing each predictor’s effects (blue vertical lines) separately. The exam output response is displayed in green lines within the confidence bounds (in red). It is noted that subject-command affected the exam output more sharply as compared to the slacking variation in exam performance caused by conditioned subject-comprehension. An interaction plot (Fig. 9) has been configured to determine which of the two predictors affected the digital exam output to a larger extent. It is indicated through the plot that an increase in subject-comprehension through online medium had influenced 22% (bold blue dot) of the exam performance. However, an exam performance would increase by 72% (bold blue dot) if the command in a subject through traditional and digital mode of delivery were to be augmented [15].

It is gathered from bi-analytic—descriptive and computational— observations that the decline in electronic-exam performance of students is relatively correlated to an abrupt transformation of learning mode without development of an objective online framework.

## Conclusion

A bi-analytic approach has been adopted to gauge students’ feedback towards attending virtual programs and how the variation of delivery medium affected their subject grasp. This discursive study also aims at determining the future course of action pertaining to what mode of instruction should be adopted or modified to optimize the learning benefits. For descriptive analysis, data visualization approach has been used to express the participants’ responses through pie and bar charts. The obtained numeric results imply that a majority of students felt discontented at not being able to achieve optimized learning outcomes post-virtual-attendance of study programs. The factors of Internet discontinuity, financial limitations, lack of technological skills’ training and an absence of computing infrastructure were deemed to compromise their experience. They communicated that the absence of regular interaction with instructors & peers partially contributed to their lack of confidence for an in-depth subject understanding. This investigation also points to the conflict of some students, especially the participants of physical sciences virtual programs, of managing and improving the subject understanding while striving to learn about online platforms’ tech language and their functioning. Moreover, a significant majority of students were observed to favor physical mode of education subsequently to fully augment the benefits of classroom learning and hands-on experience.

For computational evaluation of the data, an interaction-regression approach was utilized. An indication of blended influence of online learning and partial subject-command on unsatisfactory exam-performance was resulted. It was demonstrated through the interaction plot that obstacles in learning through digital platforms and uncertain subject-command substantially influenced virtual-exam output. Interestingly, it was estimated that 72% of the improved virtual-assessment performance was attributed to not only learning through uncomplicated online mediums, but also to how well the subject matter was understood and practiced.

To conclude, this investigation highlights the importance of automated need-based adaptations to online learning programs. It is maintained that programmed alteration to electronic learning platforms compatible with smooth manipulation of physical sciences’ content will bring a significant change in this regard. It is also imperative to design virtual guiding sessions and allow their access to students to ease the participation. Moreover, maximizing the efforts to accentuate the interaction between students and teachers through online medium can contribute to strengthening the comprehensive subject-command.

## Limitations of the study

A combination of descriptive and computational techniques was opted to present the current analysis. The related data was collected from a large batch of students of BS physical science and humanity programs of local universities.

Some limitations of the study have been highlighted here.

- Despite the rigorous selection of representative sample of students, a reflection of spontaneous and superficial judgment of experiences is inevitable in the responses, preventing a hundred percent objective estimation of data results.
- The effort was made to obtain responses from a diverse set of students to enhance the spectrum of current study. However, multiple circumstantial factors including differences in content being delivered, difficulty level of subject and various instructional designs can possibly interfere with authentic responses of questions in the survey.
- The use of statistical analysis methods and machine learning schemes is rendered to produce efficient evaluation of the data. However, minor deflection of results is possible due to use of different measures and algorithms employed to appraise the data.
- The scope of current results can always be enhanced and generalized with the inclusion of more participants from diverse background.
- The association of deteriorating virtual-assessment performance and compromised subject-command post-online-training was suggested by current data analysis. However, it must be taken into account that the relation between assessment performance and learning through physical/virtual medium is not linear, and is susceptible to vary due to several factors.

In order to augment the scope of present analysis, it is intended to further include questions assessing the role of behavioral and circumstantial factors in hampering the optimized learning through virtual platforms in the future study. Simultaneously, it is planned to incorporate deep learning schemes for the analysis of large predictors-response assessment data.

## Data Availability

The data that supports the findings of this study is available from the corresponding author, [NS] upon a reasonable request.
